# LiquidCNA: Tracking subclonal evolution from longitudinal liquid biopsies using somatic copy number alterations

**DOI:** 10.1016/j.isci.2021.102889

**Published:** 2021-07-21

**Authors:** Eszter Lakatos, Helen Hockings, Maximilian Mossner, Weini Huang, Michelle Lockley, Trevor A. Graham

**Affiliations:** 1 Centre for Genomics and Computational Biology, Barts Cancer Institute, Queen Mary University of London, London, UK; 2 Centre for Cancer Cell and Molecular Biology, Barts Cancer Institute, Queen Mary University of London, London, UK; 3 Barts Health NHS Trust, St Bartholomew's Hospital, West Smithfield, London, UK; 4 School of Mathematical Sciences, Queen Mary University of London, London, UK; 5 Department of Gynaecological Oncology, Cancer Services, University College London Hospital, London, UK

**Keywords:** Genomics, Cancer systems biology, Cancer

## Abstract

Cell-free DNA (cfDNA) measured via liquid biopsies provides a way for minimally invasive monitoring of tumor evolutionary dynamics during therapy. Here we present liquidCNA, a method to track subclonal evolution from longitudinally collected cfDNA samples sequenced through cost-effective low-pass whole-genome sequencing. LiquidCNA utilizes somatic copy number alteration (SCNA) to simultaneously genotype and quantify the size of the dominant subclone without requiring B-allele frequency information, matched-normal samples, or prior knowledge on the genetic identity of the emerging clone. We demonstrate the accuracy of liquidCNA in synthetically generated sample sets and *in vitro* mixtures of cancer cell lines. *In vivo* application in patients with metastatic lung cancer reveals the progressive emergence of a novel tumor subpopulation. LiquidCNA is straightforward to use, is computationally inexpensive, and enables continuous monitoring of subclonal evolution to understand and control-therapy-induced resistance.

## Highlights

• LiquidCNA tracks the size of the dominant subclone from longitudinal cfDNA samples
• We utilize copy number profiles from cost-efficient low-pass sequencing
• We jointly analyze all samples to robustly identify subclone-associated changes

## Graphical abstract

**Figure:**
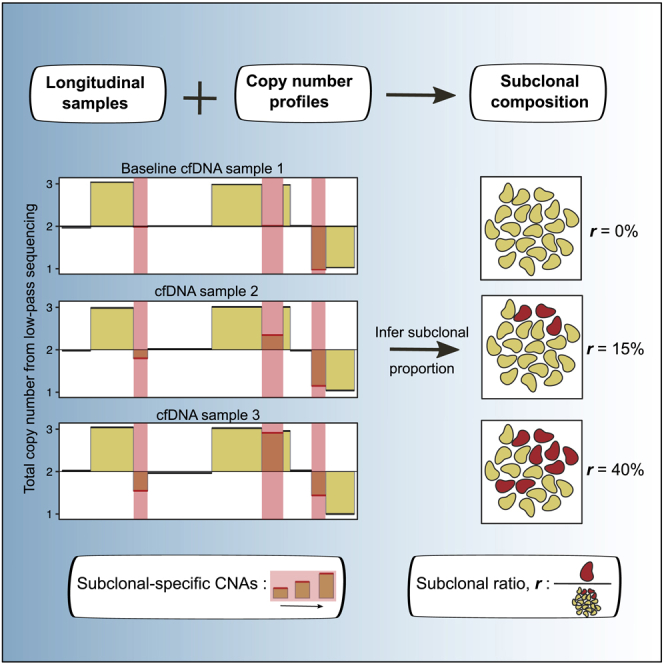

## Introduction

Liquid biopsies, primarily the analysis of cell-free DNA (cfDNA) present in blood samples, offer the potential for regular longitudinal and minimally invasive monitoring of cancer dynamics (Siravegna et al., 2017; Ng et al., 2017; Rothwell et al., 2019; Khan et al., 2018; Fernandez-Garcia et al., 2019; Conteduca et al., 2020; Nakamura et al., 2020). Circulating cfDNA is released into the blood via apoptosis or necrosis of cells. Tumor-derived cfDNA in the blood is detectable from tumors as small as 50 million cells (Diaz et al., 2012), shows correlation with disease stage (Bettegowda et al., 2014; Newman et al., 2014), and offers the same diagnostic potential as tissue-based biopsies (Nakamura et al., 2020). cfDNA is an aggregate of DNA shed from multiple locations and multiple malignant cells across the body, and hence, a single sample can provide a comprehensive overview of systemic disease. Consequently, cfDNA is an exceptional resource for noninvasive tracking of tumor composition and for monitoring response to therapy or clinical relapse.

Typically, cfDNA analysis has focused on the detection of driver gene single-nucleotide variants (SNVs), using panel-capture deep sequencing with the size of mutation-bearing clones inferred from the relative sequencing read count at the mutation site. For instance, in high-grade serous ovarian cancer (HGSOC), the frequency of *TP53* mutation in cfDNA is a measure of tumor burden and is predictive of treatment response (Parkinson et al., 2016). In colorectal cancer, *KRAS* mutation frequency in cfDNA is predictive of response to anti-EGFR therapy (Khan et al., 2018).

Somatic copy number alterations (SCNAs) and aneuploidy are widespread in cancers (Beroukhim et al., 2010; Hanahan and Weinberg, 2011; Sansregret et al., 2018; Douville et al., 2020) and have been used extensively to track tumor composition and dynamics over time (Li et al., 2014; Hieronymus et al., 2018; Rubin et al., 1992). SCNAs can be evaluated with high precision using low-pass whole-genome sequencing (lpWGS) where genome-wide sequencing is typically performed to 0.001-1X. An lpWGS library can be produced and sequenced for approximately one-tenth of the cost of a capture-based library and is robust to low sample input quality (Chin et al., 2018). A matched normal is not typically required to confirm the somatic status of detected CNAs (Scheinin et al., 2014), making rapid, high-throughput analysis of liquid biopsies by lpWGS feasible and affordable in a clinical setting (Chen et al., 2019; Adalsteinsson et al., 2017; Van Roy et al., 2017; Hovelson et al., 2017; Vanderstichele et al., 2017; Taylor et al., 2016).

However, deriving quantitative information on the proportion of tumor population that carries a particular SCNA is challenging. Tumor cells are not the only contributors to the measured DNA pool, especially in liquid biopsies where healthy DNA is often at high proportion. The measured SCNA profile is a noisy compound function of the relative tumor cell contribution to the total cfDNA pool and the specific copy number (CN) of each alteration. There are multiple sophisticated deconvolution algorithms addressing this problem in solid-tissue biopsies (Fischer et al., 2014; Yu et al., 2016; Ha et al., 2014; Oesper et al., 2014; Zaccaria and Raphael, 2020); however, these require the combined information of relative depth and B-allele frequency of a genomic region for best performance in distinguishing between concurrent models of subclonal SCNA profiles. Therefore, they are only applicable to samples sequenced to an adequate depth and paired with a matched-normal reference, while methods designed for lpWGS have been limited to estimating the tumor fraction of cfDNA samples (Adalsteinsson et al., 2017; Hovelson et al., 2017). In addition, most methods – with the notable exception of ‘deep sequencing methods’ cloneHD (Fischer et al., 2014) and HATCHet (Zaccaria and Raphael, 2020) – analyze samples independently and therefore would not explore the full breadth of information available in a longitudinal cfDNA series.

Here we present a new method to identify and track tumor subclonal evolution from longitudinal cfDNA samples based solely on lpWGS measurement of SCNAs. Our algorithm, named **liquidCNA**, determines the contribution of tumor DNA to the total cfDNA pool (i.e. cellularity/purity) and then jointly analyzes longitudinal samples of the same patient to characterize and quantify the size of the most pervasive (putatively resistant) subclone emerging or contracting over time. The method is robust enough for use in highly unstable genomes where rapid ongoing accrual of SCNAs could confound other methods and in situations where variation in sequencing coverage between samples introduces additional noise in SCNA measurement. The efficacy of the method is demonstrated using synthetic datasets, *in vitro* cell line mixtures, and *in vivo* via longitudinal analysis of cfDNA from patients with lung cancer who are undergoing treatment.

## Results

### Emergent subclone tracking from CN information

First, we derive a mathematical definition of the problem of tracking an emergent (putatively resistant) tumor subclone from longitudinal cfDNA samples, typically taken throughout the course of treatment. We consider a tumor cell population undergoing continuous evolution characterized by two cell types, namely, ancestral tumor cells (*A*) and an emerging subclone (*S*). We assume that liquid biopsies contain DNA originating from ancestral and subclonal tumor cells, as well as contaminating DNA from normal cells (*N*). The proportion of DNA arising from cells of the emergent subclone within the tumor is expressed by the subclonal ratio, *r*_*i*_, while the overall proportion of tumor-originating DNA is termed the *purity* or tumor fraction of the sample, denoted by *p*_*i*_.

**Figure 1:**
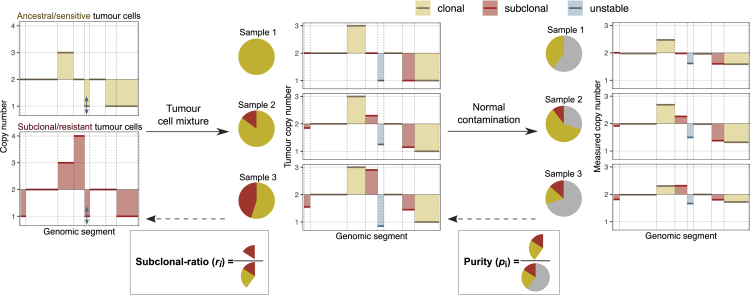
Schematic of copy number measurements The first panel shows the SCNA profile of ancestral (in yellow) and subclonal (in red) tumor cells. At different sampling time points, the overall tumor SCNA profile is a mixture of these profiles (second panel), influenced by the composition of tumor-derived DNA depicted on the pie charts. Clonal, subclonal, and unstable segments are indicated in yellow, red, and blue, respectively. Note that the CN of clonal segments remains the same. In the liquid biopsies taken at each time point, contamination from normal cells leads to ’flattened’ measured SCNA profiles (last panel) due to normal cells having a diploid karyotype. This contamination affects the CN of each segment. Our aim is to estimate purity (*p*_*i*_) and subclonal ratio (*r*_*i*_) based on clonal and subclonal SCNAs.

We consider that the CN profile of each sample has been measured – for example, using lpWGS – and so the genome can be divided into *segments*, contiguous regions of constant CN. Each measured segment CN in sample *i* ($C_{i}^{j}$) is the combination of each cell population's CN at the *j*^th^ genomic location (2 for normal cells, and *C*(*A*) and *C*(*S*) for ancestral and subclonal tumor cells, respectively), weighted by the proportions of the three populations (Figure 1). Thus, we have

(Equation 1) $$C_{i}^{j}=2+p_{i}\left(\left(1-r_{i}\right)C{\left(A\right)}^{j}+r_{i}C{\left(S\right)}^{j}-2\right).$$

We assume that each segment can fall into one of the three categories depending on its CN in ancestral and subclonal tumor cells. *Clonal* alterations (and unaltered segments) are at the same CN in both tumor populations, and their measured CN is only affected by the purity of a sample. *Subclonal* segments represent SCNAs that are unique to the emerging subclone. Their measured CN is influenced by the subclonal ratio of a sample, as well as sample purity. Note that these SCNAs might appear clonal in a single time point – in the extreme case of the emerging population overtaking the entire tumor – but not in all samples. Finally, segments that do not follow either of these patterns – owing to uncertain measurements or ongoing instability – are termed *unstable*. Our aim is to estimate the underlying purity and subclonal ratio, *p*_*i*_ and *r*_*i*_, from longitudinal CN measurements of clonal and subclonal segments (Figure 1).

### Estimation of subclonal ratio

**Figure 2:**
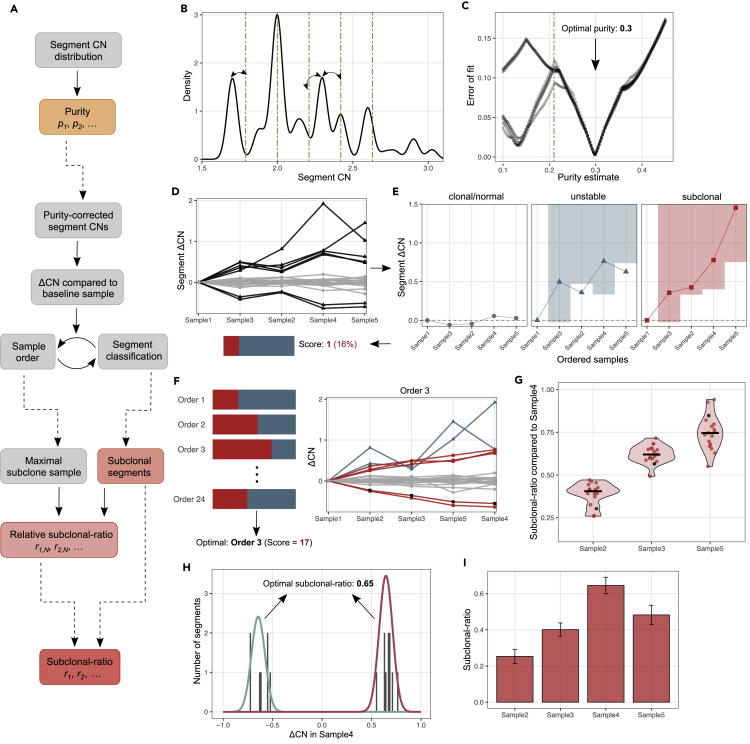
Illustration of the estimation algorithm (A) Outline of the estimation algorithm, with estimation outputs highlighted in color. Dashed arrows separate independent modules. (B) Purity estimation based on the peaks of the distribution of segment CNs. Green lines show the peaks expected at an example purity of 0.21. (C) The error of a range of purity estimates, computed from the distance of observed and estimated peaks in (B). Each line corresponds to a smoothing kernel applied to the raw segment CN distribution. The optimal purity is indicated with arrows. (D) Change in segment CN values (ΔCNs) plotted according to an example sample order. The number of subclonal segments computed in (E) is indicated below. (E) Classification of segments based on the sample order in (D). Segments with low variance are classified as clonal (gray). Nonclonal segments are evaluated whether they follow a quasi-monotone pattern (indicated by the shaded regions) and classified as unstable (outside of shaded region, blue) or subclonal (red). (F) ΔCN values plotted according to the optimal sample order maximizing subclonal segments. Line colors indicate the class of each segment as in (E). (G) Relative subclonal ratio estimation compared with maximal subclonal ratio sample (rightmost in (F)). Points show individual segment-wise estimates, with an example segment highlighted in black. Black line shows the median. (H and I) Subclonal ratios and confidence intervals inferred by fitting a Gaussian mixture model to the ΔCN distribution of subclonal segments. The components of the best fit with means −*r* and *r* are shown in green and magenta, respectively, in (H).

Estimation is carried out in three steps (Figure 2A and STAR Methods). First, the *purity* of each sample is assessed using the distribution of segment CN values. We assume that the majority of segments have integer CN in all tumor cells; hence, the distribution is expected to have distinct peaks at regular intervals of *p*_*i*_, corresponding to clonal segments with CN of 1, 2, 3, etc (Figure 2B). We derive the purity estimate as the value that minimizes the squared error between observed and expected peaks (Figure 2C). The inferred purity values are used to correct the segment CN values, thus estimating the tumor-specific CN of each segment.

LiquidCNA does not require a mainly diploid tumor genome (i.e. major peak at CN = 2) to derive correct estimates but will derive erroneous conclusions if the CN values – as measured by the CN quantification software, e.g. QDNAseq (Scheinin et al., 2014) – are incorrectly centered (e.g. major peak is defined as CN 2, but the true value is CN 3). To control for this, an initial manual check of the CN profile is recommended prior to applying liquidCNA and renormalization to the correct ploidy if required.

Next, for every segment, we compute the change in CN, ΔCN, between each sample and a *baseline* sample that is assumed to have negligible proportions of the emerging (putatively resistant) subclone – for example, a sample taken upon diagnosis or before the start of therapy. ΔCN values naturally highlight subclone-associated segments altered in nonbaseline samples, as these segments display markedly positive (CN gain compared with baseline) or negative (CN loss) values (Figure 2D). From these ΔCNs, we then establish the set of segments that are subclonal and the sample ordering that reflects increasing subclonal proportions. To do this, we examine each possible order of samples, classifying each segment as clonal (if the variance of its ΔCNs across samples is below a predefined threshold), subclonal (if it shows monotone change in ΔCN value along the order of the samples – i.e. if the ΔCNs are consistent with an emerging subclone), or unstable (if it does not correlate with sample order) according to that order (Figure 2E). The order with the highest proportion of segments classified as subclonal is selected, and these subclonal segments are used for downstream computation of tumor composition (Figure 2F). The methodology ensures that the *dominant* subclone associated with the most pervasive SCNAs is evaluated and that subclonal ratio inference is robust to segments with unstable CN.

Finally, we compute the relative and absolute subclonal ratio of each sample using the identified set of subclonal segments. Relative subclonal ratios are defined as the median ratio of segment ΔCNs compared with the sample with the maximum subclonal proportion (Figure 2G). The absolute subclonal ratio is computed based on the assumption that subclonal segment ΔCN values correspond to distinct SCNAs that differ between ancestral and subclonal cells. The subclonal ratio of sample *i* is therefore derived as the shared mean (*r*_*i*_) of a mixtures of Gaussian distributions with constrained means −*r*_*i*_,+*r*_*i*_, etc., fitting the ΔCN distribution of subclonal segments (Figure 2H). We also provide the 95% confidence interval of the absolute subclonal ratio estimate based on the shared variance of the fitted Gaussians (Figure 2I).

LiquidCNA outputs both relative and absolute subclonal ratio measures, because for most applications the relative value holds sufficient information on how the subclonal (putative resistant) population changes between time points. Relative proportions are also less susceptible to the measurement noise in the measured segment CNs, while a combination of low subclonal proportion and high sequencing noise can cause the fitting of absolute subclonal ratio estimates to fail to converge.

### Synthetic mixed populations

**Figure 3:**
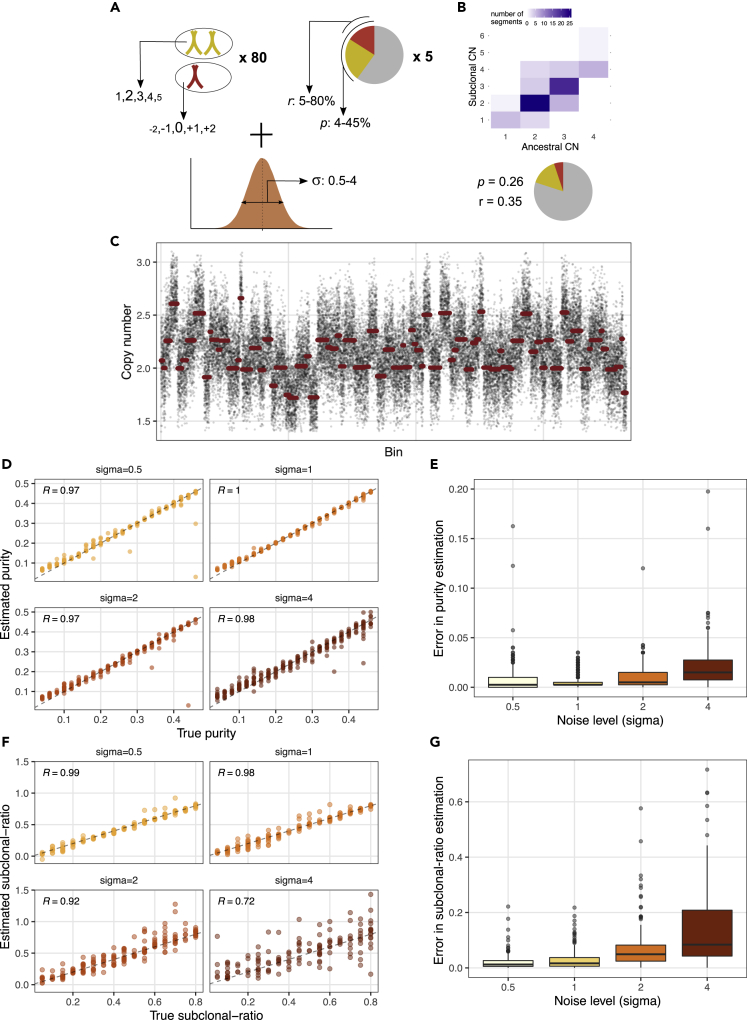
Estimation of mixtures of synthetic cell populations (A) Parameters used to randomly sample synthetic datasets including simulated measurement noise. The font size of copy number states indicates their probability. (B) A randomly generated sample. The heatmap depicts the distribution of segment CNs in ancestral and subclonal cells, and the proportion of cell populations is shown on the pie chart (red: subclonal, yellow: ancestral, gray: normal). (C) Copy number profile of the sample in (B), with raw bin-wise and segmented copy number values shown in black and red, respectively. (D) Estimated purity of 1,000 synthetic samples with varying levels of noise (σ), plotted against the true theoretical purity. The *y* = *x* line is indicated with dashes. (E) Error of purity estimation (absolute difference to true purity) for samples with noise level indicated on the x axis. (F) True and estimated subclonal ratios of 200 synthetic datasets (1,000 samples) with varying levels of noise (σ). (G) Error in subclonal ratio estimation for datasets with increasing noise level. Box plot elements in (E) & (G) stand for the following: center line, median; box limits, upper and lower quartiles; whiskers, 1.5x interquartile range; points, outliers. *R* in (D) & (F) indicates the Pearson correlation coefficient; *p* < 10^−8^ for all panels. See also Figures S1–S3.

We first evaluated the performance of liquidCNA using *synthetic* datasets where input values of subclonal proportion and purity were known. We generated these datasets matching characteristics of measurements from patients, and therefore, each dataset is equivalent to a longitudinal *synthetic experiment*, typically consisting of 4–5 synthetic samples. In order to simulate imperfect measurements, we added varying levels of normally distributed measurement noise (defined by the dimensionless parameter σ) to bin-wise CN values (Figures 3A–3C and STAR Methods).

We evaluated the accuracy of the purity estimation on 1000 synthetic samples (from 200 experiments/datasets, Figure 3D) and found that purity *p* could be estimated within 2% of the true tumor fraction in 90% of samples at noise levels *σ* ≤ 1. The error on the purity estimation was greater when the noise was increased (Figure 3E) and was most pronounced in samples with high noise and low tumor fraction.

Next, we derived subclonal ratios using purity-corrected CN profiles on synthetic mixtures with purity ≥0.1. We set a threshold to filter out clonal segments (see Figure 2E) such that at least 10 segments were retained and the proportion of retained segments classified as subclonal was maximal following segment classification. Figure 3F shows the true and estimated subclonal ratios for 50 synthetic experiments. Overall, we found that the subclonal ratio was estimated with ~5% error, and the accuracy decreased with higher measurement noise (Figure 3G). Relative subclonal ratios (calculated compared with the sample with highest subclonal proportion) were estimated with higher accuracy (error ~3%, Figures S1A and S1B). We found that computing absolute subclonal ratios in a two-step process from these values yielded similar results to direct estimation by fitting a Gaussians mixture model and provided an estimate even in cases where the direct estimation did not converge (Figure S1C and STAR Methods).

We also evaluated if a higher proportion of unstables segments decreased accuracy, but found that, unlike noise, it had little effect on the estimation accuracy (Figure S2). Finally, we explored how the overall number of samples (measurement time points) influenced subclonal ratio estimation. As expected, datasets with only 2 measurements (consisting of a baseline and single nonbaseline sample) were estimated with reduced accuracy since unstable and subclonal segments could not be distinguished in this case. Accuracy increased with the number of samples available for evaluation and showed negligible improvement above 4 samples (Figures S3A and S3B). Relative subclonal ratio on the other hand could be estimated with high accuracy at all sample numbers (Figures S3C and S3D).

### Mixtures of ovarian cancer cell lines

Next, we evaluated liquidCNA on lpWGS data derived from *in vitro* mixtures of two paired high-grade serous ovarian cancer (HGSOC) cell lines (Hoare et al., 2020) (see STAR Methods and Table S1). HGSOC cells were ideally suited for this evaluation as high levels of chromosomal instability are a hallmark of the disease (Nelson et al., 2020; Cancer Genome Atlas Research Network, 2011). We anticipated that liquidCNA will be most applicable for the tracking of subclonal evolution in malignancies with high CNA burden (Vanderstichele et al., 2017).

**Figure 4:**
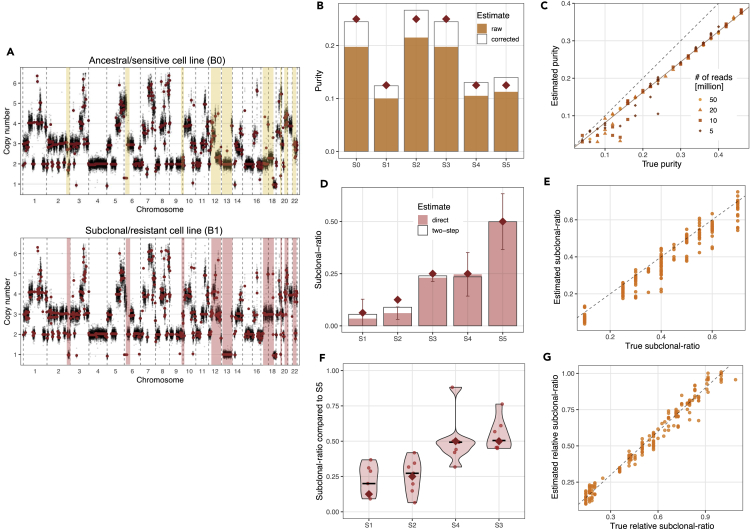
Estimation of mixtures of high-grade serous ovarian cancer cell lines (A) Copy number profile of the ancestral/sensitive and subclonal/resistant HGSOC cell lines. Raw bin-wise and segmented copy number values are shown in black and red, respectively. Resistant-specific subclonal SCNAs are highlighted. (B) Purity estimates of samples S0-S5. Corrected values are computed using the linear fit in (C). Theoretical purity values are indicated by maroon diamonds. (C) True (theoretical) and estimated tumor purity of 120 *in silico* HGSOC cell line mixtures. *y* = *x* and the linear fit of the estimates (*y* = 0.81*x*) are shown with dashed and solid lines, respectively. Point shape and shade indicate the total number of reads per sample. (D) Subclonal ratio estimates for samples S1-S5. Shaded and empty bars indicate estimates derived using direct (Gaussian fit) and two-step (from relative ratios in (F)) methods, respectively. Error bars show 95% confidence interval of the direct estimate, and maroon diamonds indicate theoretical values. (E) True and estimated subclonal ratios of 50 *in silico* datasets constructed of samples from (C) with 50 million reads. (F) Relative subclonal ratio estimates for samples S1-S4, compared to S5. Estimates from each subclonal segment are shown with dots, the median estimates are indicated by black lines, and true values are indicated by maroon diamonds. (G) True and estimated relative subclonal ratios in the 50 datasets shown in (E). See also Figures S5–S8.

We divided a population of OVCAR4 cells into an untreated ‘sensitive/ancestral’ and a ‘resistant/subclonal’ aliquot, the latter cultured to develop resistance to platinum-containing chemotherapy. In addition to the high SCNA burden inherited from the ancestral sensitive cell line, resistant cells acquired new SCNAs during the *in vitro* evolution of resistance (Figure 4A). In varying known proportions, we then mixed the genomic DNA extracted from the two cell lines and further diluted the mixtures with DNA from blood samples of healthy volunteers assumed to have a diploid genome (Table S1). These DNA mixtures were sequenced to mean depth 1.3x, and composite SCNA profiles were generated using QDNAseq (Scheinin et al., 2014) (see STAR Methods). In addition, we generated further *in silico* mixtures by sampling and mixing genome-aligned reads from sequencing data from each of the three cell types sequenced individually. In these mixtures, we controlled the total number of reads per sample to study the effect of variable read depth and associated measurement noise.

First, we used liquidCNA to estimate the purity of *in vitro* mixed samples (samples S0-S5). The purity of each sample was estimated to be lower than the theoretical mixing proportion (Figure 4B). In the *in silico* mixed samples, we found that there was a strong linear relationship between estimated and true purity (Figure 4C). The underestimation of purity is explained by our definition of theoretical purity in the *in vitro* and *in silico* mixing procedure (defined as proportion of DNA weight versus the proportion of read counts, respectively). A highly aneuploid genome has a higher weight than a diploid genome; therefore, mixing of equal weights results in a higher proportion of normal genomes than expected. Our purity estimates were in agreement with observed peaks of the CN distribution (Figure S4A), and by fitting a linear model to the estimates, the theoretical tumor fraction could be fully recovered, as illustrated by the ‘corrected’ estimates of samples S0-S5 (Figure 4B). The number of reads (sequencing depth) did not systematically influence the accuracy of estimating tumor fraction, but purity estimates of samples with low tumor fraction were noisier at low read depth (Figure 4C). As samples below 10% tumor fraction are estimated with lower accuracy and are more influenced by noise, we advise to complement purity estimation with orthogonal measures or manual curation or discard such samples from downstream analysis.

Next, we inferred the subclonal ratio for cell line mixtures using purity-corrected ΔCN values, with sample S0 used as the baseline sample for both *in vitro* and *in silico* sample sets. We could correctly order cell line mixtures according to subclonal ratios without any *a priori* information (Figure S4B), and both absolute subclonal ratio and relative subclonal changes were estimated on average within 2% and 3% of the true subclonal percentage (Figures 4D and 4F). In particular, we note that samples S4 and S3 were accurately estimated as having an equal subclonal ratio, despite originating from different biological replicates with different tumor purity. We also note that even though there were no *truly unstable* segments in this dataset as measurements were not taken over time, three nonclonal segments were classified as such, owing to relatively small size and higher noise in their measured CN value.

Using datasets of randomly selected *in silico* samples with 50 million reads, we confirmed that our algorithm could accurately infer the subclonal ratio of samples, in particular when considering relative proportions (Figures 4E and 4G). Although the estimation quality decreased with lower read counts (Figure S5), in most cases the estimated absolute and relative subclonal ratios were within 15% and 10% of the true subclonal proportion, respectively. Furthermore, we found that cases with high estimation error were typically caused by misestimation of purity of low-purity samples, which could be easily identified and removed without *a priori* information, as demonstrated in Figure S6.

Using the known theoretical mixing values of tumor DNA content – instead of data-derived estimates – to derive purity-corrected CN values increased the estimation error, especially in low-read-count samples (Figure S7). This finding emphasizes that nondiploid genomes might bias alternative measurement methods and that internal consistency in the method of deriving sample characteristics (purity and subclonal ratio) is crucial when assessing the dynamics of the subclonal population.

We further validated our results using ichorCNA, a recent bioinformatic method designed specifically for screening the tumor fraction of low-coverage cfDNA samples with high sensitivity (Adalsteinsson et al., 2017). IchorCNA uses a hidden Markov model and Bayesian inference framework to tackle the problem of accurately estimating tumor purity in samples sequenced to 0.1x coverage (Adalsteinsson et al., 2017). In addition to normal contamination, ichorCNA also derives an estimate on subclone fraction – but it is important to note that the algorithm was not optimized for subclonal deconvolution. We applied ichorCNA to both *in vitro* and *in silico* samples to derive purity and subclonal ratio estimates, the latter corrected for the fact that ichorCNA has no knowledge of the subclone's identity as ancestral/subclonal (see STAR Methods). We found that our purity results derived by liquidCNA were consistent with the estimates from ichorCNA. As ichorCNA offered a higher accuracy in cases with low purity and read count, we believe this method could be used to ‘save’ these cases (e.g. S108 in Figure S6) for downstream analysis. On the other hand, we found that liquidCNA substantially outperformed ichorCNA in estimating the subclonal ratio since our tool performs a specialized joint analysis of all patient samples (Figure S8).

### Subclonal analysis of patient samples

We used liquidCNA to analyze emergent subclones in longitudinal cfDNA samples from patients with non-small cell lung cancer (NSCLC) undergoing therapy, as previously reported by Chen et al. (2019). The liquid biopsies were collected as part of the FIGARO study (GO27912, NCT01493843), a randomized phase II trial designed to evaluate the efficacy of pictilisib, a selective inhibitor of phosphatidylinositol 3-kinase (Soria et al., 2017). Pictilisib or placebo was given in combination with standard chemotherapy regimen which was determined based on the subtype of NSCLC. Blood samples were taken at baseline (day 1 of the first treatment cycle) and at 6-week intervals up to the end of treatment (EOT). DNA was isolated from the plasma of liquid biopsies and sequenced using lpWGS to an average depth of 0.5x, as described in detail in the study by Chen et al., 2019.

Chen et al. (2019) identified several SCNAs in EOT samples that were absent at baseline and described several genes within these regions that might be associated with resistance. We sought to apply liquidCNA to these cases to corroborate their observations and further to quantify the size of emergent subclones over time in these patients.

We obtained the lpWGS data (fastq files) and performed CN profiling (see STAR Methods) on patients with cfDNA samples from ≥3 time points (*n* = 32). We identified three patients (1306, 2760, and 3209) whose sample series fulfilled the following criteria: (i) had a cfDNA sample taken on the first day of therapy with purity above ~20% and (ii) had at least two nonbaseline samples with purity above ~20%. Patients 1306 and 3209 were in the experimental arm of the study, while patient 2760 was assigned to the control arm; all three patients have progressed during the course of the trial.

**Figure 5:**
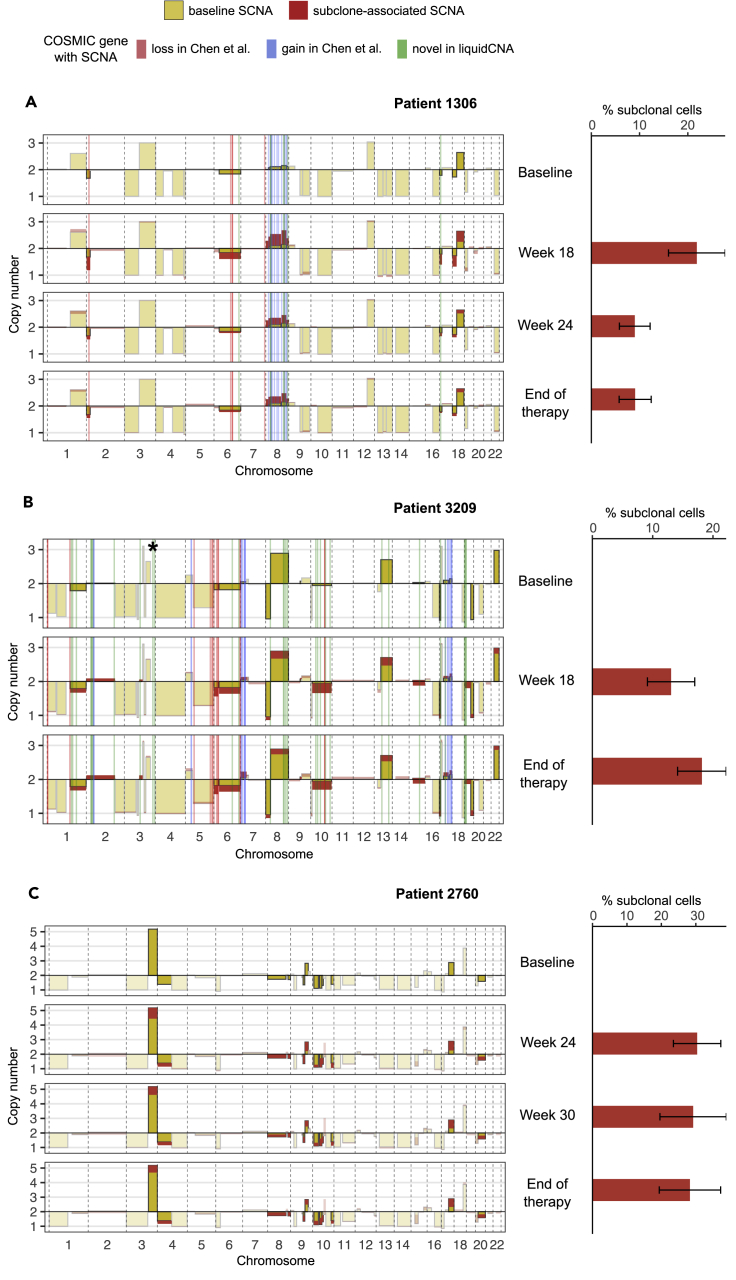
Estimation in cfDNA samples from patient data Subclone-specific copy number changes and subclonal ratio in lung cancer patients 1306 (A), 3209 (B), and 2760 (C) from the study by Chen et al., 2019. Left: purity-corrected SCNA profiles. Yellow bars show the CN of each segment in the baseline sample, and red bars indicate subclonal deviations from this value in nonbaseline samples. Regions of subclone-specific CNAs are also indicated by darker colors. Shaded regions indicate the location of putatively therapy-associated cancer genes identified in the original study with CN losses (in red) and CN gains (in blue) and newly identified in liquidCNA (in green, see also Figure S10). A bar of CN > 8 on chromosome 3 (indicated by asterisk) has been omitted from (B) for better visualization. Right: estimated subclonal proportion of each sample with 95% confidence intervals. Note that only samples with >10% purity were analyzed (see also Figure S9) and patient 2760 had no gene annotation in the study by Chen et al., 2019.

We ran liquidCNA on data from these selected patients, discarding samples with purity below 10% (Figure S9), and examined the genomic segments that liquidCNA identified as subclonal relative to baseline samples (Figure 5). We also examined genes from the Catalog of Somatic Mutations in Cancer (COSMIC, Sondka et al. (2018)) Cancer Gene Census that were located in subclonal regions (see STAR Methods) and compared with the list of genes identified by Chen et al as carrying therapy-specific SCNAs (Figure 5 and S8 of Chen et al., 2019).

We observed a good overlap with the gene set reported by Chen et al., with 15 of 16 and 24 of 35 therapy-associated genes recovered by liquidCNA in patients 1306 and 3209, respectively (Figures S10A and S10B). The direction of subclonality (loss or gain) aligned with the original study's findings for all 39 subclonal genes. In patient 1306, only gene MLL3 (on chromosome 7) was not identified by liquidCNA, which we believe was a highly focal loss missed owing to the large bin size (500 kb) used in CN calls. In patient 3209, we found three gene clusters (8/35 genes, located on 1p, 5p and 5q) that were classified as clonal rather than subclonal by liquidCNA. We found that SCNAs in these regions were reported in all samples (including baseline) in the original study as well; although ΔCN from baseline was too small for liquidCNA to declare these segments subclonal, the direction (sign) of ΔCN was in agreement with the original study. Three of 35 genes fell into genomic regions that were filtered out during the quality control step, owing to small size (PDE4DIP on chromosome 1) or inconsistent read mapping (IRF4 and TRIM27 on chromosome 6).

We also identified 7 and 32 “novel” subclonal genes not highlighted by Chen et al., 2019 in patients 1306 and 3209, respectively (in green in Figure 5 and S10A-B). The majority of these were in regions near previously identified genes, confirming that the discrepancy between the two studies is due to different SCNA bin sizes. However, we also found a subclonal loss in patient 1306 (TP53 on chromosome 17) and multiple losses in patient 3209 (chromosome 8 and 13), which had nondiploid CN in the baseline samples; therefore, these did not pass the filtering criteria of the original study, but could be identified with liquidCNA.

Overall, we found that liquidCNA could reliably identify subclone-associated SCNAs but provided conservative datasets of therapy-associated genes. We believe its sensitivity can be improved by appropriate choice of bin size in case the data quality allows and detecting all candidate genes is of highest importance. We also note that subclone-associated SCNAs might be “passengers” in the subclone and are not necessarily enriched for genes with functional impact on therapeutic response, and therefore, subclone-associated driver genes should be regarded only as candidates.

Finally, we quantified the size of the emerging subclone and found that it accounted for 10 to 30% of the tumor-derived DNA in the cfDNA in the three patients evaluated (Figure 5). Patient 2760 showed evidence of a subclonal proportion consistently around 30%, which could be explained by samples from this patient taken at later time points (24-36 weeks after the start of therapy). Patient 1306, on the other hand, showed a contracting subclone that reduced in proportion from 20% presence at week 18 to <10% at the end of therapy. Samples from patient 3209 obtained at 18 weeks and at the end of therapy contained less than 20% DNA derived from subclonal tumor cells. However, this patient had only 3 samples passing quality control, which we showed can lead to less accurate estimates (Figure S3). To explore accuracy in patient samples, we repeated subclonal ratio estimation for patients 1306 and 2760 on only the last two time points and found the estimates were consistent (Figure S10C), confirming that subclonal proportions for patient 3209 are robust. Overall, we could track subclonal evolution and in case the total population size was known – which might be accessible from additional measurements of the tumor-associated cfDNA pool – the tumor subclone fractions established here could also be converted into growth rates to enable future predictions of the tumor dynamics.

## Discussion

We present liquidCNA, a computational algorithm to infer subclonal dynamics using low-coverage sequencing measurements of aneuploidy in cfDNA samples. Our algorithm performs joint analysis of multiple longitudinal samples to identify sample purity, subclonal SCNAs, and the abundance of an emerging subclone. LiquidCNA has been designed to work on depth-of-coverage CN information with low-pass whole genome sequenced and liquid biopsies in mind, where matched normal baseline or single-nucleotide polymorphisms required for most contemporary deconvolution algorithms are not available. Furthermore, liquidCNA utilizes joint analysis of samples from the same patient to distinguish SCNAs associated with the emerging subclone and those showing unstable behavior and consequently is robust against uncertain SCNA measurements.

We validate our method, both on synthetic SCNA datasets and *in vitro* and *in silico* mixtures of two ovarian cancer cell lines. We successfully infer the proportion of the dominant subclone in all of the aforementioned datasets, with good accuracy across a range of sample qualities defined by the noise level or sequenced reads. We compare our results with ichorCNA, a state-of-the-art method for the analysis of low-coverage cfDNA samples, and show that liquidCNA achieves similar accuracy in estimating tumor purity, while also offering accurate reconstruction of subclonal structure, not available from other methods. In patients with lung cancer, liquidCNA applied to lpWGS data derived from longitudinal cfDNA shows the emergence of subclones during therapy and identifies genomic regions associated with the emergent tumor cells.

We demonstrate that liquidCNA can identify and quantify emerging subclones from cfDNA samples, therefore enabling tracking of tumor subclone evolution through the course of therapy. Deciphering the evolutionary trajectory of cancers can aid prognostic and therapeutic decision-making and further our understanding of therapy-induced drug resistance. Measuring the dynamics of tumor composition is particularly crucial for prospective monitoring during an adaptive therapy regime aiming to control resistant subclones (Gatenby et al., 2009; Enriquez-Navas et al., 2015; Zhang et al., 2017). Furthermore, the proportion of cfDNA that is tumor-derived (what we term ’purity’) in itself is a promising biomarker for determining initial therapy response and prognosis (Fernandez-Garcia et al., 2019; Choudhury et al., 2018), as well as for tracking tumor progression during and after therapy (Ng et al., 2017; Newman et al., 2014; Conteduca et al., 2020; Chen et al., 2019).

In summary, we provide a robust tool to derive quantitative information about dynamic changes in clonal composition from SCNA measurements derived from low-pass whole-genome sequenced cfDNA samples. LiquidCNA enables cost-efficient real-time noninvasive tracking of subclonal tumor evolution, which can provide new insights into the evolution of SCNAs and the dynamical emergence of therapy-associated resistance.

### Limitations of study

We note that there are limitations in our method, which should be taken into account when designing experiments to be analyzed using liquidCNA.

Since our inference relies on heterogeneous CN profiles (>10% genome altered) and subclone-specific SCNAs (at least 3-5 segments), we cannot analyze cancer (sub)types with very low chromosomal instability, for example, microsatellite unstable tumors. Conversely, extremely high levels of ongoing instability might bias our analysis owing to the lack of a stable subclone-associated SCNA profile. Therefore, liquidCNA is not suitable for oligo-metastatic disease if spatially separate metastases carry distinct karyotypes. Similarly, liquidCNA might give biased results if longitudinal samples are taken very sparsely, when enough time elapses for clonal sweeps and substantial karyotype changes (e.g. due to novel metastases) that our assumption of a smooth evolutionary trajectory does not hold anymore. We also note that liquidCNA tracks a *single* dominant subclone associated with the largest set of subclone-specific SCNAs, and consequently, if there are multiple smaller subclones (with less or no associated SCNAs), these will be ignored by the algorithm. Similarly, the emerging subclonal karyotype identified by liquidCNA does not necessarily represent an evolutionary advantage, and therefore, we urge caution in interpreting it.

The accuracy of our estimation reduces at low purity, as purity-corrected CN values, and consequently subclonal ratio estimates are sensitive to inaccuracy in purity estimation. Furthermore, the accuracy of our estimation decreases with sequencing depth (increased noise), leading to a higher probability of misestimated purity values if a sample has less than 10 million sequencing reads. Therefore, we advise to (i) further curate purity estimates when possible and (ii) discard samples below ~10% purity, or <20% for samples sequenced only to 5 million reads or lower. While this limits the number of samples, tumor fractions above this regime were observed in a substantial number of patients, especially in late-stage disease where liquidCNA can offer the largest benefit (Chen et al., 2019; Adalsteinsson et al., 2017; Conteduca et al., 2020; Bettegowda et al., 2014; Hovelson et al., 2017; Phallen et al., 2017). In addition, recent studies have shown that the unique fragment length of tumor-derived cfDNA can be utilized to enrich tumor purity either experimentally or bioinformatically (Mouliere et al., 2011, 2018; Underhill et al., 2016).

Taken together, we believe liquidCNA is most suitable for applications where (blood) samples are taken frequently – e.g. during prognostic monitoring – and sequenced to a depth to produce at least 10-15 million reads per sample. Such periodic samples ensure that between-time-point instability is minimal and that an appropriate number of samples are available even following strict quality and purity control, while obtaining >10 million reads of multiple samples is still possible at minimal cost.

We also note that although liquidCNA could potentially be used to track the emergence of subclonal population(s) under therapy, our *in vitro* validation did not test liquidCNA in such a setting. For this potential application to be successful, we require that the emerging populations are uniquely marked by a number of SCNAs. In some cases, treatment response appears to be driven by “phenotypic plasticity” where cellular behavior changes without underlying genetic change (Turati et al., 2021; Shaffer et al., 2017). This scenario violates a key assumption of liquidCNA that the emerging population is dominated by a subclone of distinct karyotype, and so we would expect the application of liquidCNA to fail. While liquidCNA could provide useful insights in tumor/treatment scenarios where SCNAs are known to dominate evolution during treatment, other scenarios should be handled with caution, and the limitations of liquidCNA in an evolution-of-resistance setting will have to be evaluated in future studies.

## STAR★Methods

### Key resources table

**Table:** 

REAGENT or RESOURCE	SOURCE	IDENTIFIER
**Critical commercial assays**		

NEBNext Ultra II kit	New England Biolabs	Cat# E7645S
NovaSeq 6000	Illumina	RRID: SCR_016387

**Deposited data**		

Raw sequencing data of HGSOC cell line mixtures	This study	ENA: PRJEB42332
Copy number call data from *in vitro* and in silico mixtures	This study	https://github.com/elakatos/liquidCNA_data
Human reference genome hg19	Genome Reference Consortium	http://hgdownload.soe.ucsc.edu/goldenPath/hg19/bigZips/
Cancer driver genes	COSMIC Cancer Gene Census, Sondka et al. (2018)	https://cancer.sanger.ac.uk/census
Genomic location of genes	Ensembl BioMart	https://www.ensembl.org/biomart/martview

**Experimental models: cell lines**		

Human: Sensitive HGSOC cell line	Laboratory of Prof Fran Balkwill	HGSOC cell line OVCAR4
Human: resistant HGSOC cell line	Laboratory of Dr Michelle Lockley, Hoare et al. (2020)	HGSOC cell line Ov4Cis

**Software and algorithms**		

LiquidCNA	This study	https://github.com/elakatos/liquidCNA
Scripts for generating and analyzing synthetic mixtures	This study	https://github.com/elakatos/liquidCNA
QDNAseq	Scheinin et al., 2014	https://bioconductor.org/packages/release/bioc/html/QDNAseq.html
ichorCNA	Adalsteinsson et al. (2017)	https://github.com/broadinstitute/ichorCNA

**Other**		

Sequencing data of cfDNA samples from the FIGARO trial	Chen et al. (2019)	Available from the original authors

### Resource availability

#### Lead contact

Further information and requests for resources and reagents should be directed to and will be fulfilled by the lead contact, Trevor Graham (t.graham@qmul.ac.uk).

#### Material avalability

This study did not generate any new unique reagents.

### Experimental model and subject details

HGSOC cell line OVCAR4 was obtained from Prof Fran Balkwill (Barts Cancer Institute, UK) and grown in Dulbecco's Modified Eagle Medium (DMEM) containing 10% fetal bovine serum (FBS) and 1% penicillin/streptomycin. A resistant/subclonal HGSOC cell line (Ov4Cis) was generated by culturing an aliquot of the ancestral OVCAR4 cell line in increasing concentrations of cisplatin. For further details on cell culture and the cell lines, refer to the study by Hoare et al., 2020.

We then extracted genomic DNA from both cell lines and from blood samples from healthy volunteers using QIAamp DNA Micro Kit (Qiagen, Hilden, Germany). Genomic DNA from the three sources was mixed in varying proportions (Table S1), measured as the mass of DNA inputted from each source, to a total of 20 ng of DNA per sample, and subjected to sonication using the Covaris M220 system. Libraries were prepared using the NEBNext Ultra II kit (New England Biolabs, Hitchin, United Kingdom) with 4 cycles of PCR amplification, indexed with unique dual-indexing primers, and sequenced on Illumina NovaSeq 6000 to a mean depth of 1.3x.

### Method details

#### CN measurements

We consider a tumor that consists of two distinct cell populations, ancestral (*A*) and subclonal (*S*) tumor cells, and continuously sheds cfDNA into the blood circulation. A typical scenario would be ancestral cells representing drug-sensitive tumor cells present before cancer therapy and subclonal cells denoting the emerging subclone with resistance to therapy. The proportion of DNA originating from these two cell types changes over time as we take measurements via blood samples (Figure 1). Since cfDNA found in blood can also originate from normal (nontumor) cells of the body, the measured DNA is contributed by a mixture of the two tumor cell populations (*A* and *S*) and normal cells (*N*). At each time point *i*, the proportion of these three populations in the measured sample, *s*_*i*_, depends on the proportion of all tumor-derived DNA (the *purity* of the sample, *p*_*i*_) and the proportion of subclone-derived DNA from the tumor (the subclonal ratio, *r*_*i*_):

(Equation 2) $$N_{i}=1-p_{i};\;A_{i}=p_{i}\cdot \left(1-r_{i}\right);\;S_{i}=p_{i}\cdot r_{i}.$$

Our aim is to track the dynamics of the subclonal (putatively resistant) population by determining the subclonal ratio for each time point, *r*_*i*_ or the change in subclonal ratio between time points, *r*_*i*_/*r*_*k*_ = *r*_*ik*_. To this end, we use the CN values as typically measured by lpWGS of the sequential cfDNA samples.

Let us consider distinct genomic regions with homogeneous CN state, *segments*. We assume that the CN state of most segments stays constant over time in a particular population. Therefore, the *jth* segment is characterized by a set of three time-independent absolute CN states, *C*(*N*)^*j*^,*C*(*A*)^*j*^,*C*(*S*)^*j*^, corresponding to the local CN in normal, ancestral, and subclonal cells, respectively. The CN of segment *j* as measured in the *ith* sample, $C_{i}^{j}$, is the combination of these three absolute CNs, weighted by the proportions of DNA derived from the three cell populations at that time point (*N*_*i*_, *A*_*i*_, *S*_*i*_). We know that normal cells are at a diploid state, and hence, *C*(*N*)^*j*^ = 2 for all *j*. Therefore, using the purity and subclonal ratio defined in Equation (2),

(Equation 3) $$C_{i}^{j}=2+p_{i}\left(\left(1-r_{i}\right)C{\left(A\right)}^{j}+r_{i}C{\left(S\right)}^{j}-2\right).$$

Since all cells in a cell population share the absolute CN for a given segment, the values *C*(*S*)^*j*^ and *C*(*A*)^*j*^ are always integers. Therefore, in theory, measured CNs from a given sample should be limited to a discrete set of values defined by these integer states, making it possible to solve the set of equations formed by Equation (3) for *p*_*i*_ and *r*_*i*_ using linear algebra.

However, we have to take into account that all real sequencing measurements have a level of imprecision introducing variation on top of this relationship. Using the term *σ*_*ij*_ to represent the noise in the *i*th measurement of segment *j*, Equation (3) becomes

(Equation 4) $$C_{i}^{j}=2+p_{i}\left(\left(1-r_{i}\right)C{\left(A\right)}^{j}+r_{i}C{\left(S\right)}^{j}-2\right)+\sigma_{ij}.$$

with the magnitude and family of this noise depending on the specifics of the technology used for CN measurement, especially the sequencing depth (Scheinin et al., 2014). This measurement noise – associated with a continuous distribution – broadens the set of $C_{i}^{j}$ values, rendering a linear algebra solution impossible. Hence, our aim becomes to derive an inference of *p*_*i*_ and *r*_*i*_ despite this unknown noise, $\sigma_{ij}$.

#### Segment classification

Each segment can fall into three categories depending on their respective CN states in the two types of cells. (i) *Clonal* segments have the same absolute CN in ancestral and subclonal tumor cells, *C*(*A*)^*j*^ = *C*(*S*)^*j*^. A special case of clonal segments are segments of *normal* (diploid) CN, where *C*(*A*)^*j*^ = *C*(*S*)^*j*^ = 2. (ii) *Subclonal* segments have different absolute CNs in the ancestral and subclonal tumor population, *C*(*A*)^*j*^≠*C*(*S*)^*j*^. These segments represent SCNAs that distinguish the *subclone* from its ancestor, even though they are not necessarily associated with a selective/phenotypic difference (e.g. drug resistance) directly. (iii) *Unstable* segments are neither clonal nor associated with the emergent subclone and therefore are best described by a time-dependent tumor-wide CN value, $\zeta {\left(T\right)}_{i}^{j}$, that does *not* depend on *r*_*i*_. These segments can arise if a genomic region cannot be measured reliably or if ongoing genomic instability introduces novel SCNAs during the time tracked by our samples. We can assume that the number of such segments is small compared with the total number of measured segments.

Depending on whether segments are clonal, subclonal, or unstable, their measured CN across samples will change according to the subclonal ratio and purity of each sample:

(Equation 5) $$C_{i}^{j}=2+p_{i}\left(C{\left(A\right)}^{j}-2\right),\;\text{if the segment is }clonal,$$

(Equation 6) $$C_{i}^{j}=2+p_{i}\left(C{\left(A\right)}^{j}-2+r_{i}\left(C{\left(S\right)}^{j}-C{\left(A\right)}^{j}\right)\right),\;\text{if the segment is }subclonal,$$

(Equation 7) $$C_{i}^{j}=2+p_{i}(\zeta {\left(T\right)}_{i}^{j}))$$

For simplicity, we omit the term *σ*_*ij*_ and its derivatives, but the reader should keep in mind that all equations are subject to measurement noise. Figure 1 illustrates how the measured CN of segments depends on the parameters *r*_*i*_ and *p*_*i*_ highlighted above. In the following sections, we use Equations (5) and (6) to estimate the underlying parameters, *p*_*i*_ and *r*_*i*_, via three steps (Figure 2).

#### Purity estimation

Purity estimation is carried out based on clonal (including normal/diploid) segments. In general, we expect the majority of segments to fall into this category. Consequently, for the majority of segments, their measured CN follows Equation (5). Since *C*(*A*)^*j*^ can take only integer values, the distribution of segment CNs is expected to have distinct peaks at regular intervals of *p*_*i*_.

Using a peak-finder algorithm on the smoothed distribution of measured CN values, we directly compare the peaks to the values expected at a given purity, $\left\{2-p_{i},2,2+p_{i},2+2p_{i},\ldots \right\}$, as shown in Figure 2B. The error of the fit to a purity, *p*_*i*_, is evaluated as the summed squared distance between each peak and the closest observed peak,

(Equation 8) $${\underset{C\left(A\right)}{\sum }\text{min}((2+p_{i}(C(A)-2))-\text{peaks})}^{2})$$

As the detected peaks of the data depend on the smoothing kernel used on the distribution, we perform this computation for a wide range of smoothing bandwidths (0.5×−2.5× the default value) and derive the purity estimate, ${\hat{p}}_{i}$, as the value that minimizes the mean and/or median error across the range (Figure 2C).

Then, we use the derived ${\hat{p}}_{i}$ to renormalize the measured CN values and thus eliminate normal contamination. We gain an estimate of the tumor-specific CN ($C{\left(T\right)}_{i}^{j}$), a mixture of ancestral and subclonal CNs:

(Equation 9) $$\hat{C}{\left(T\right)}_{i}^{j}=\frac{1}{{\hat{p}}_{i}}\cdot \left(C_{i}^{j}-2\right)+2\approx C{\left(A\right)}^{j}+r_{i}\left(C{\left(S\right)}^{j}-C{\left(A\right)}^{j}\right).$$

Note that, due to the noise in measurements, peaks from close absolute CNs can become indistinguishable in low-purity samples. Therefore, we expect purity values below 5% to be indistinguishable (unless high-sequencing depth is available) and also advise to discard samples with low purity (typically *p*_*i*_ < 0.1) as erroneous purity estimations can bias downstream computation.

#### Identifying subclonal segments

Next, we aim to identify the subset of segments with subclone-specific *subclonal* SCNAs that reflect the changes in subclonal ratio over time. To easily assess the *change* in segment CNs, we designate a sample as *baseline* and compute the change in segment CN, ΔCN, between each sample and this baseline sample. Typically, the sample taken upon diagnosis or before the start of therapy (usually the first time point, *s*_1_) can be used. We can assume that this sample has no or only negligible population of the emerging subclone and therefore represents a pure ancestral population:

$$r_{1}\approx 0\to C{\left(T\right)}_{1}^{j}\approx C{\left(A\right)}^{j}.$$

Hence, the change in CN of a subclonal segment compared with the baseline becomes

(Equation 10) $$\text{Δ}C{\left(T\right)}_{i}^{j}=C{\left(T\right)}_{i}^{j}-C{\left(T\right)}_{1}^{j}=r_{i}\left(C{\left(S\right)}^{j}-C{\left(A\right)}^{j}\right).$$

Furthermore, Equation (10) provides an informative quantity even if the baseline sample is not pure, as $\text{Δ}C{\left(T\right)}_{i}^{j}$ nonetheless describes the *change* in subclone-specific SCNAs.

In order to uncover which segments are truly *subclonal*, and how the subclonal ratio changes over measurements, we need to identify a pervasive pattern across samples, and the subset of segments that consistently follows it. If the samples were taken so that the subclonal population increases over time points, this pattern would be a monotone increase or decrease for all segments with subclone-specific SCNAs. While we cannot assume that the samples are taken in order of increasing subclonal proportions (e.g. a change of treatment between sampling times might lead to fluctuating population size in a resistance-associated subclone), we can aim to rearrange them to follow this rule.

Consequently, we rephrase our aim as deriving (i) a *set of subclonal segments* that follow a monotone pattern across ordered samples and (ii) an *ordering of samples* that is correlated with by the maximum number of (subclonal) segments. Formally, we are looking for a subset of segments, $\left\{j_{1},j_{2},\ldots \right\}$, and a permutation of samples (starting from the designated baseline sample), $s_{1},s_{i},\ldots ,s_{N}$, where for every segment. $j\in \left\{j_{1},j_{2},\ldots \right\}$

either

(Equation 11) $$\begin{array}{l} \text{Δ}C{\left(T\right)}_{i+1}^{j}-\text{Δ}C{\left(T\right)}_{i}^{j}>-\varepsilon ,\;\forall i\\ \;or\\ \text{Δ}C{\left(T\right)}_{i+1}^{j}-\text{Δ}C{\left(T\right)}_{i}^{j}<\varepsilon ,\;\forall i \end{array}$$

holds for all *i* for a predefined accuracy level, *ε*. We use an ε>0 accuracy level to allow for samples with near-equal subclonal ratio measured with uncertainty. We find that for typical lpWGS datasets, ε≈0.02−0.05 works well to account for the underlying measurement noise.

Figures 2D–2F illustrate the derivation of optimal sample order and subclonal segment set. We first separate clonal segments: since these have relative CN values of 0, apart from some measurement noise, we filter out any segment that has a standard deviation below a predefined threshold. We then evaluate Equation (11) over all remaining segments and over all orderings of the samples. As we expect 4-6 time points per dataset, an exhaustive search of all possible permutations is feasible. Given a permutation, each segment is classified according to whether it follows Equation (11) – these are candidate subclone-specific and unstable segments, respectively (Figure 2E). The optimal sample order is defined as the permutation that maximizes the number of subclonal segments (Figure 2F).

#### Subclonal ratio estimation

Finally, we use the set of segments identified as *subclonal* and compute the subclonal ratio of each time point. We derive the (absolute) subclonal ratio, *r*_*i*_, for each sample using Equation (10). As both *C*(*A*)^*j*^ and *C*(*S*)^*j*^ are assumed to be integers, and we know that $C{\left(A\right)}^{j}\neq C{\left(S\right)}^{j}$,

(Equation 12) $$\text{Δ}C{\left(T\right)}_{i}^{j}\in \left\{\ldots ,-2r_{i},-r_{i},r_{i},2r_{i},\ldots \right\},\;\forall \;j\in \left\{j_{1},j_{2},\ldots \right\}.$$

To take into account that the measured ΔCNs compared with the baseline, $\text{Δ}\hat{C}{\left(T\right)}_{i}^{j}$, are influenced by noise, we fit these values with a mixture of Gaussian distributions where the mean of the Gaussians follows Equation (12), as illustrated in Figure 2H. The subclonal ratio of a sample is derived as the constrained mean parameter, *r*_*i*_, of the Gaussian mixture optimizing the fit (Figure 2I). The 95% confidence interval of the inferred subclonal ratio is computed based on the (shared) variance of the fitted constrained Gaussians.

The measurement noise propagated from segment CNs can lead to high spread in values, making estimates less robust and rendering the resolution of low subclonal ratios (*r*_*i*_ ≤ 0.1) challenging, occasionally leading to the Gaussian-fitting step to fail. Therefore, we also derive *relative* subclonal ratios, which allow for a more general application not limited to good-quality samples. In particular, relative values are compared with the maximal sample since its subclonal ratio is assumed to be the most robust against measurement noise. We compute the relative deviation of each normalized subclonal tumor segment CN

(Equation 13) $$\text{Δ}C_{iN}^{j}=\frac{\text{Δ}C{\left(T\right)}_{i}^{j}}{\text{Δ}C{\left(T\right)}_{N}^{j}}=\frac{r_{i}\left(C{\left(S\right)}^{j}-C{\left(A\right)}^{j}\right)}{r_{N}\left(C{\left(S\right)}^{j}-C{\left(A\right)}^{j}\right)}=\frac{r_{i}}{r_{N}},$$

giving rise to a distribution of relative subclonal ratio estimates (Figure 2G). We derive a point estimate for the relative *r*_*i*_ of each sample as the median of this set,

(Equation 14) $${\hat{r}}_{iN}=\text{median}\left(\text{Δ}C_{iN}^{j}\right),\;j\in \left\{j_{1},j_{2},\ldots \right\}.$$

Absolute subclonal ratio estimates can then be derived using these relative estimates in a two-step estimation process (as opposed to the direct estimation above): we derive $r_{N}$ based on Equation (12) and subsequently compute $r_{iN}\cdot r_{N}$ to retrieve *r*_*i*_.

#### Generating synthetic and in silico datasets

We constructed synthetic datasets of 80 segments (of length varying between 120 and 800 bins) and 5 time points (unless stated otherwise) as illustrated in Figure 3A. For each segment, we generated sensitive segment CN states (*C*(*S*)^*j*^) by randomly sampling from {1,2,3,4,5}, with diploid and close-to-diploid states occurring with higher frequency. Subclone-specific absolute CNs ($C{\left(S\right)}^{j}$) were assigned by randomly sampling from $C{\left(A\right)}^{j}+\left\{-2,-1,0,1,2\right\}$, with no change (giving rise to clonal segments) having a higher weight. For each sample, *s*_*i*_, we assigned purity and subclonal ratio randomly from the ranges 0.04 < *p*_*i*_ < 0.45 and 0.05 < *r*_*i*_ < 0.8, with the exception of the baseline samples, where *r*_1_ < 0.04. We then recreated the measurement procedure of computing noise-ridden raw CN values in a given segment, *j*, by adding a normally distributed noise. The magnitude (standard deviation) of the noise was controlled by the noise level parameter, σ (representing differences arising from, e.g. sequencing depth) and the CN of the segment (reflecting higher variance in higher CN states):

(Equation 15) $$\text{raw}C_{i}^{bin}=2+p_{i}\left(\left(1-r_{i}\right)C{\left(A\right)}^{j}+r_{i}C{\left(S\right)}^{j}-2\right)+\text{Normal}\left(0,f\left(\sigma ,C_{i}^{j}\right)\right).$$

The final CN value of each segments, ${\hat{C}}_{i}^{j}$, was computed as the mean of all raw$C_{i}^{bin}$ contained in the segment. In addition, we selected 2.5-15% of segments as *unstable* and resampled their tumor-specific CN value to be independent of *r*_*i*_. Figures 3B and 3C show parameters of a synthetic sample and its CN profile.

*In silico* mixtures were generated by bioinformatically mixing sequencing reads of DNA derived from the ancestral/sensitive, subclonal/resistant tumor cell lines and healthy blood cells. Similarly to synthetic samples, for each *in silico* sample, we randomly assigned purity, 0.1 < *p*_*i*_ < 0.45, and subclonal ratio, 0.05 < *r*_*i*_ < 0.8. We then sampled reads (using samtools view -s) from aligned read (bam) files of ‘pure’ ancestral, subclonal, and normal samples (B0, B1 and N0) in proportions to match *p*_*i*_(1−*r*_*i*_), *p*_*i*_*r*_*i*_ and 1−*p*_*i*_, respectively. We also varied the total number of reads per sample (as a proxy for sequencing depth and consequently measurement noise) and generated 30-30 samples with 50, 20, 10, and 5 million total reads each.

#### Processing lpWGS samples

Fastq files derived from lpWGS samples (generated via sequencing cell line mixtures or obtained from the study by Chen et al., 2019) were aligned to the human reference genome (version *hg19*, using bwa). We then processed bam files using the QDNAseq R package (Scheinin et al., 2014) using DNAcopy for segmentation (Venkatraman and Olshen, 2007). QDNAseq produced two CN values for each genomic bin: a *raw* presegmentation value and a *segmented* value grouping bins of equal CN together. The CN of bins on the predefined blacklist of QDNAseq and of those with <75% mappability was set to NA. Raw and segmented CN values for all cell line samples are available from https://github.com/elakatos/liquidCNA_data.

Since QDNAseq returns normalized CN values (with normal/diploid state at value 1), we multiplied all values by 2 before proceeding with the estimation algorithm and renormalized segment CN values to be centered at 2 exactly. We then redefined segment boundaries using the ensemble of samples as regions of constant CN in *all* samples. This way break points present in only a subset of samples (such as a subclone-specific SCNA) gave rise to segments handled separately for all samples. Updated segments with length below 6 mega-bases (120 bins of 50kb [cell line mixtures] or 12 bins of 500kb [patient cfDNA samples]) were excluded from the downstream analysis to filter out short segments sensitive to localized measurement biases.

Finally, we curated each segment CN by discarding bins with the most extreme 2.5% of raw segment values and recalculating the segment CN value as the mean of normal distribution fitted to the remaining raw CNs. We found that this curation had negligible effect for most segments but successfully improved assigned segment CN values for more error-prone genomic regions.

We also ran ichorCNA (Adalsteinsson et al., 2017) on the aligned bam files of cell line mixture samples. We used the snakemake workflow available from within ichorCNA, using bin sizes of 500 kb with corresponding GC and mappability files, no matched normal panel, maximum allowed CN of 6, and default parameter values otherwise. We defined ichorCNA-estimated estimates as the outputted tumor fraction value and subclonal ratio as the best estimate out of the outputted subclone fraction or (1−subclone fraction), accounting for that ichorCNA was agnostic to ancestral/subclonal identities.

#### Subclone-associated gene analysis

We extracted genomic coordinates of segments identified as subclonal from cfDNA samples from patients 1306 and 3209 of the FIGARO trial. We downloaded the latest release of the COSMIC Cancer Gene Census (Sondka et al., 2018) and retrieved gene locations for each gene using Ensembl BioMart (https://www.ensembl.org/biomart/martview). We defined genes as subclone-associated drivers that (i) fell in subclonal segments and (ii) were listed with a somatic-mutation-based association to any type of lung cancer or undefined (‘other’) cancer. We also extracted a gene list for each patient from Figure 5 and S8 of the study by Chen et al., 2019, together with the directionality each gene's SCNA (loss or gain) identified by Chen et al. We then evaluated the Δ*CN* values measured at locations of genes from the study by Chen et al., 2019 (irrespective of whether these were identified as subclonal) and expanded their list with COSMIC cancer genes ‘newly’ identified in liquidCNA. The final gene tables were used to generate heatmaps (using ggplot2) in Figure S10 and are available from https://github.com/elakatos/liquidCNA_data.

### Quantification and statistical analysis

All analyses were carried out in R (version 4.0.3) and plots created using ggplot2 (version 3.3.3). Correlation coefficients were computed using the stat_cor function from ggpubr (version 0.4.0).

## Data Availability

• Aligned sequencing data from HGSOC cell lines and *in vitro* mixtures (listed in Table S1) are available from the European Nucleotide Archive (ENA: PRJEB42332). Raw and postsegmentation CN values for these samples are available from https://github.com/elakatos/liquidCNA_data. Therapy-associated gene CNs from patients 1306 and 3209 of the FIGARO trial are also available at https://github.com/elakatos/liquidCNA_data. • Estimation functions of liquidCNA implemented in R (version 4.0.3), an illustrative example in a Jupyter notebook, and code generating and analyzing synthetic and *in silico* data are available from https://github.com/elakatos/liquidCNA. • Any additional information required to reanalyze the data reported in this paper is available from the lead contact upon request.
