# Supplementary material for: LiquidCNA: Tracking subclonal evolution from longitudinal liquid biopsies using somatic copy number alterations
Source: iScience. 2021 Jul 21;24(8):102889. doi: 10.1016/j.isci.2021.102889 (PMC8350516; doi:10.1016/j.isci.2021.102889)
Supplement: Document S1. Figures S1–S10 and Table S1 [file mmc1.pdf]

iScience, Volume *24*

Supplemental information

LiquidCNA: Tracking subclonal evolution from longitudinal liquid biopsies using somatic copy number alterations

Eszter Lakatos, Helen Hockings, Maximilian Mossner, Weini Huang, Michelle Lockley, and Trevor A. Graham

Sample	Read count	Normal	Ancestral	Subclonal
B0	98 million	0%	100%	0%
B1	76 million	0%	0%	100%
N0	82 million	100%	0%	0%
S0	87 million	75%	25%	0%
S1	80 million	87.5%	11.72%	0.78%
S2	70.5 million	75%	21.875%	3.125%
S3	102 million	75%	18.75%	6.25%
S4	83 million	87.5%	9.375%	3.125%s
S5	70.5 million	87.5%	6.25%	6.25%

Table S1: Proportion of DNA originating from normal, ancestral/sensitive and subclonal/resistant cells in the HGSOC cell line samples; related to Fig. 4. Samples S0-S5 and *in silico* mixtures of B0, B1 and N0 are analysed in Figure 4.

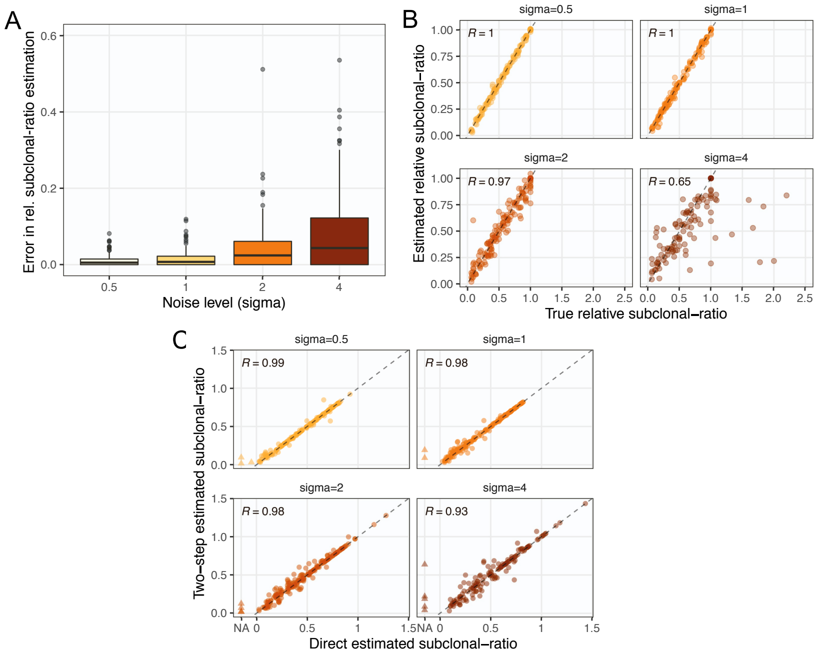

Figure S1: Subclonal-ratio estimation of synthetic mixtures using relative subclonal-ratios, related to Fig. 3. (A) Error in relative subclonal-ratio estimation (compared to the sample with the maximal subclonal proportion) for 200 datasets (1,000 samples) with increasing noise level (σ), indicated on the x axis. Box-plot elements represent: center line, median; box limits, upper and lower quartiles; whiskers, 1.5x interquartile range; points, outliers. (B) True and estimated relative subclonal-ratio of the synthetic datasets in (A). (C) Subclonal-ratio estimates of the samples in (A) computed directly from Gaussian fits (x axis) or in a two-step estimation via relative subclonal-ratios (y axis), at different levels of σ. Samples where the direct estimation failed are indicated with triangles at value NA. R in (B)&(C) indicates the Pearson correlation coefficient; $p < 10^{-8}$ for all panels.

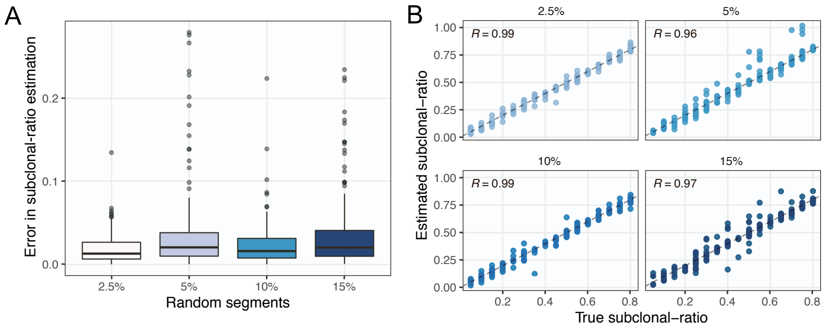

Figure S2: Subclonal-ratio estimation of synthetic mixtures with varying number of random segments, related to Fig. 3. (A) Error in the subclonal-ratio estimate of 200 synthetic datasets (1,000 samples) with $\sigma = 1$ and the proportion of segments with random CN values indicated on the x axis. Box-plot elements represent: center line, median; box limits, upper and lower quartiles; whiskers, 1.5x interquartile range; points, outliers. (B) True and estimated subclonal-ratio of the synthetic datasets in (A). R indicates the Pearson correlation coefficient; $p < 10^{-8}$ for all panels.

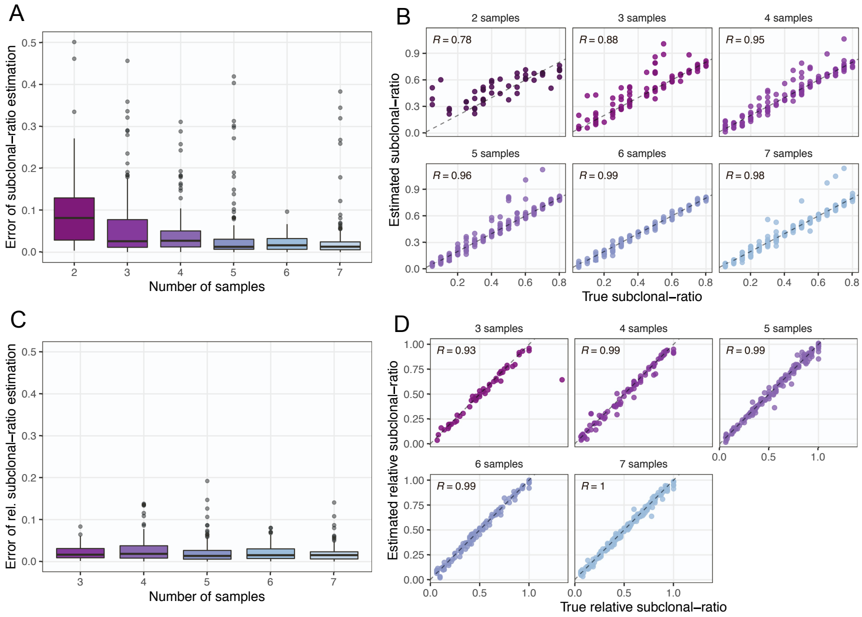

Figure S3: Subclonal-ratio estimation of synthetic mixtures from datasets of varying sample number, related to Fig. 3. (A,C) Error in the absolute (A) and relative (C) subclonal-ratio estimation of 300 synthetic datasets with $\sigma = 1$ and the number of samples in the dataset indicated on the x axis. Box-plot elements represent: center line, median; box limits, upper and lower quartiles; whiskers, 1.5x interquartile range; points, outliers. (B,D) True and estimated subclonal-ratio of the synthetic datasets in (A) and (C), respectively. R indicates the Pearson correlation coefficient; $p < 10^{-8}$ for all panels.

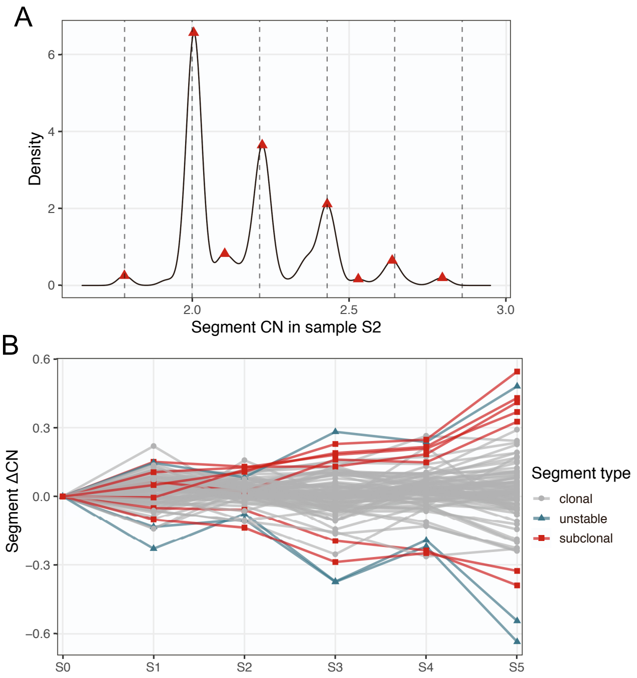

Figure S4: Estimation details of HGSOC cell line mixtures, related to Fig. 4. (A) The distribution of measured segment CN values in sample S2. Peaks used in purity estimation are highlighted by red dots. Dashed vertical lines indicate the expected peak locations at the best fitting purity estimate, $\hat{p}_{S2} = 0.215$. (B) ΔCN values of samples S0-S5, compared to the baseline sample, S0. Samples are shown in the order associated with increasing subclonal proportion, and coloured according to segment class (grey: clonal, blue: unstable, red: subclonal).

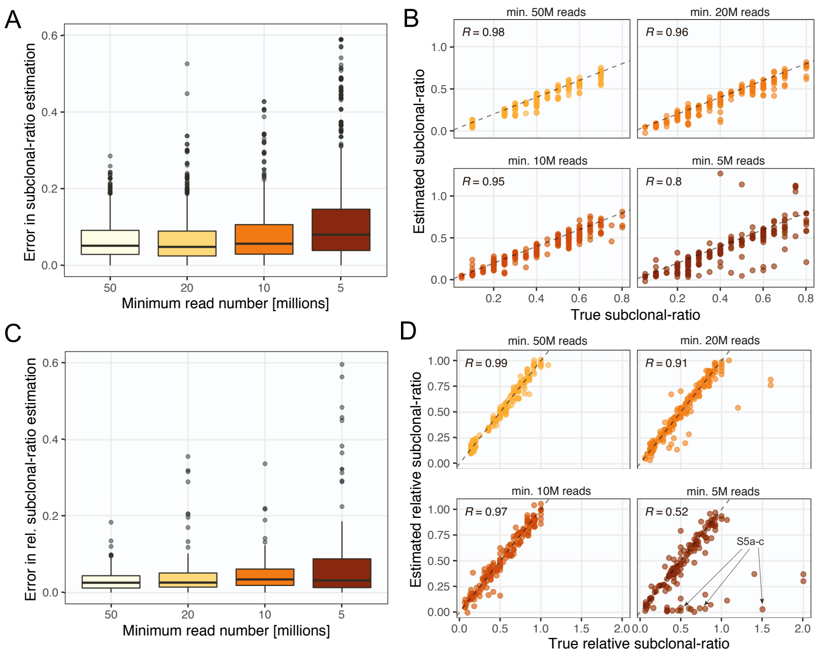

Figure S5: Subclonal-ratio estimation of *in silico* cell line mixtures with varying read counts, related to Fig. 4. (A,C) Error in absolute (A) and relative (C) subclonal-ratio estimation for 200 datasets constructed of *in silico* samples with minimum required read count indicated on the x axis. Box-plots represent: center line, median; box limits, upper and lower quartiles; whiskers, 1.5x interquartile range; points, outliers. (B,D) True and estimated absolute (B) and relative (D) subclonal-ratio of the synthetic datasets in (A) and (C). R indicates the Pearson correlation coefficient; $p < 10^{-8}$ for all panels. The dataset indicated by arrows is re-analysed in Figure S6.

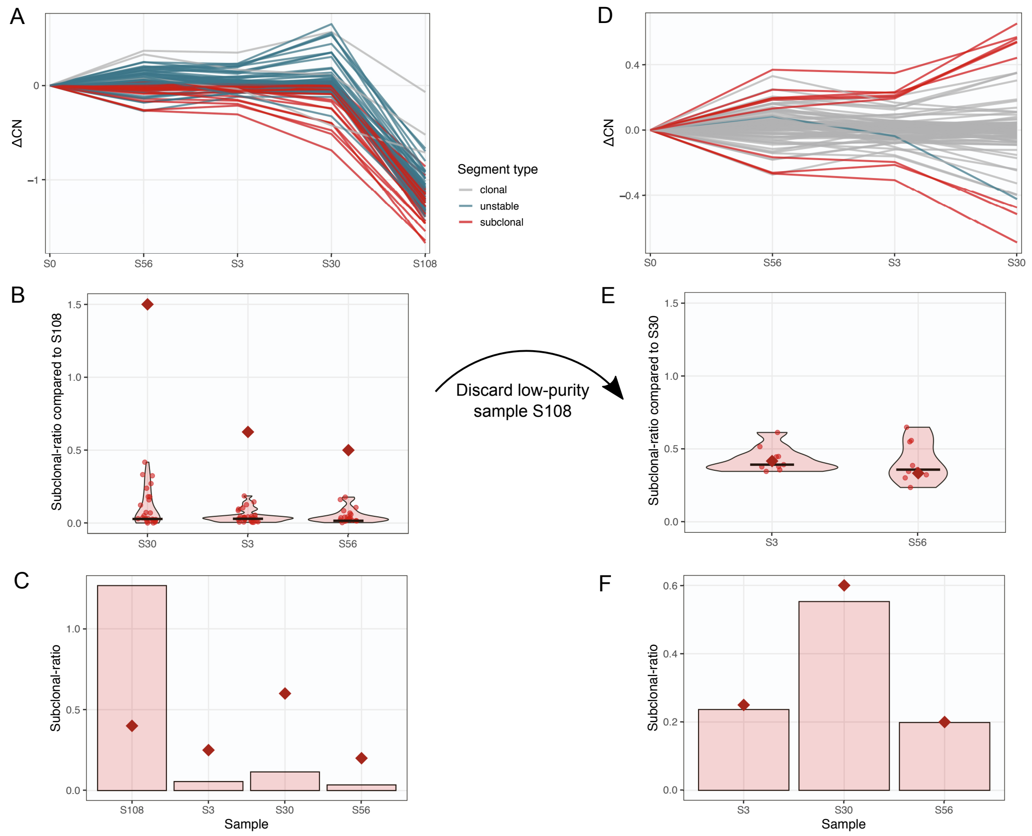

Figure S6: Manual curation of in silico cell line mixture dataset, related to Fig. 4. (A,D) ΔCN values and classification with automatically assigned ordering of samples (A). As sample S108 clearly shows incorrect patterns due to mis-estimated (and low) purity, it is discarded from downstream analysis (D). (B,E) Relative subclonal-ratio distribution computed for the original (B) and curated (E) maximal sample. Estimates from each segment are shown with dots, the median estimates are indicated by black lines, the true relative ratios with maroon diamonds. (C,F) Absolute subclonal-ratio estimates of the original (C) and curated (F) datasets. True values are indicated with maroon diamonds.

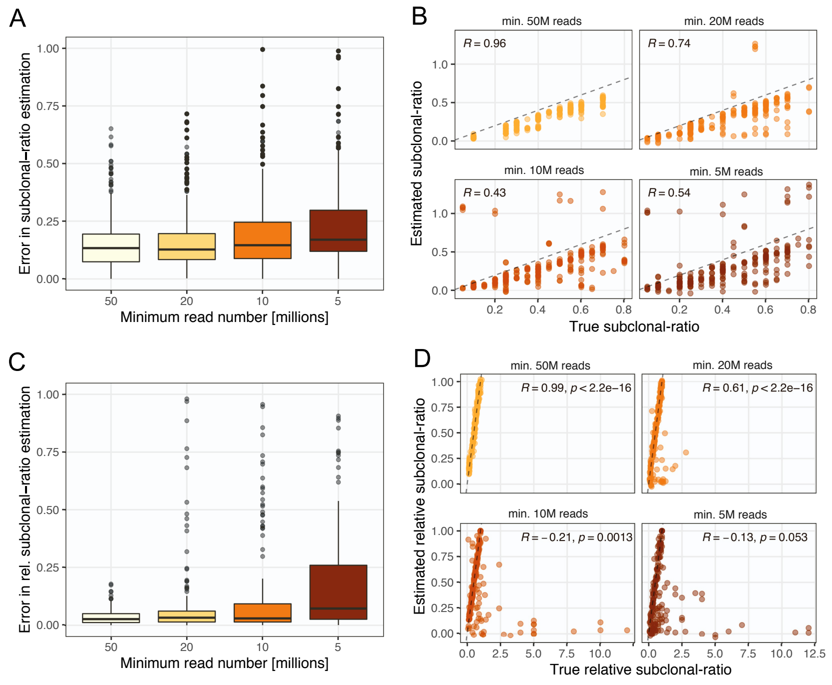

Figure S7: Subclonal-ratio estimation of *in silico* cell line mixtures, with CN values of each sample corrected using the theoretical rather than estimated tumour purity value; related to Fig. 4. (A,C) Error in absolute (A) and relative (C) subclonal-ratio estimation of 200 datasets with minimum required read count indicated on the x axis. Box-plots represent: center line, median; box limits, upper and lower quartiles; whiskers, 1.5x interquartile range; points, outliers. (B,D) True and estimated subclonal-ratio of the datasets in (A) and (C), respectively. R indicates the Pearson correlation coefficient; $p < 10^{-8}$ for all panels in (B).

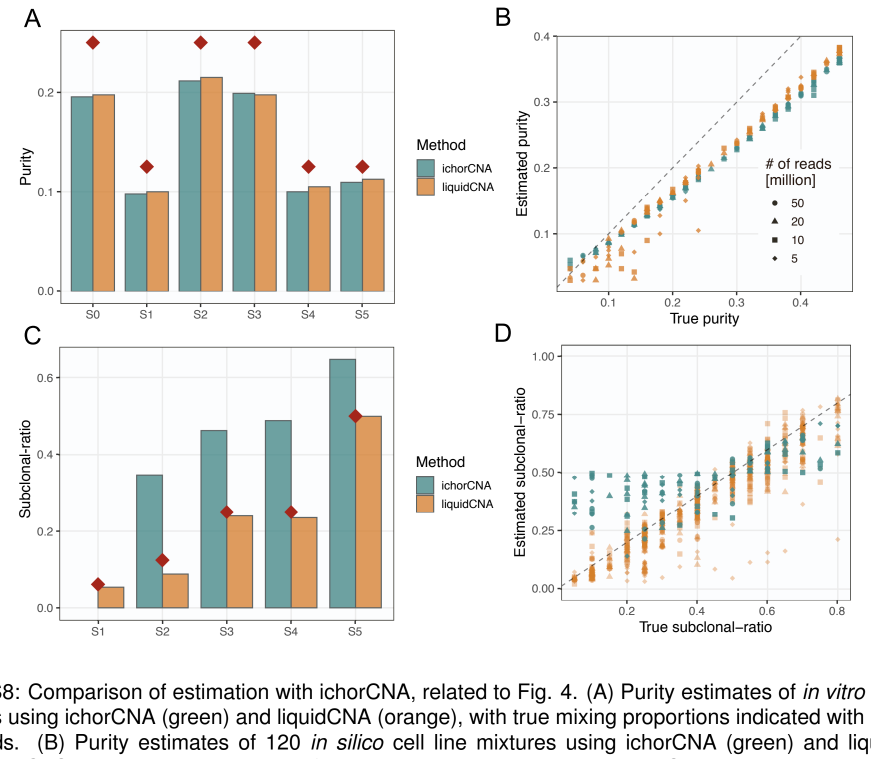

Figure S8: Comparison of estimation with ichorCNA, related to Fig. 4. (A) Purity estimates of *in vitro* cell line mixtures using ichorCNA (green) and liquidCNA (orange), with true mixing proportions indicated with maroon diamonds. (B) Purity estimates of 120 *in silico* cell line mixtures using ichorCNA (green) and liquidCNA (orange). (C) Subclonal-ratio estimates of *in vitro* cell line mixtures using ichorCNA (computed as corrected subclonal fraction, in green) and liquidCNA (computed via the two-step method, in orange), with true mixing proportions indicated with maroon diamonds. (D) Subclonal-ratio estimates of*in silico* cell line mixtures using ichorCNA (in green, corrected to provide best results) and liquidCNA (in orange, using randomly sampled datasets) (cf. Fig. S5). Point shapes indicate the minimum number of reads in each sample, as shown in (B).

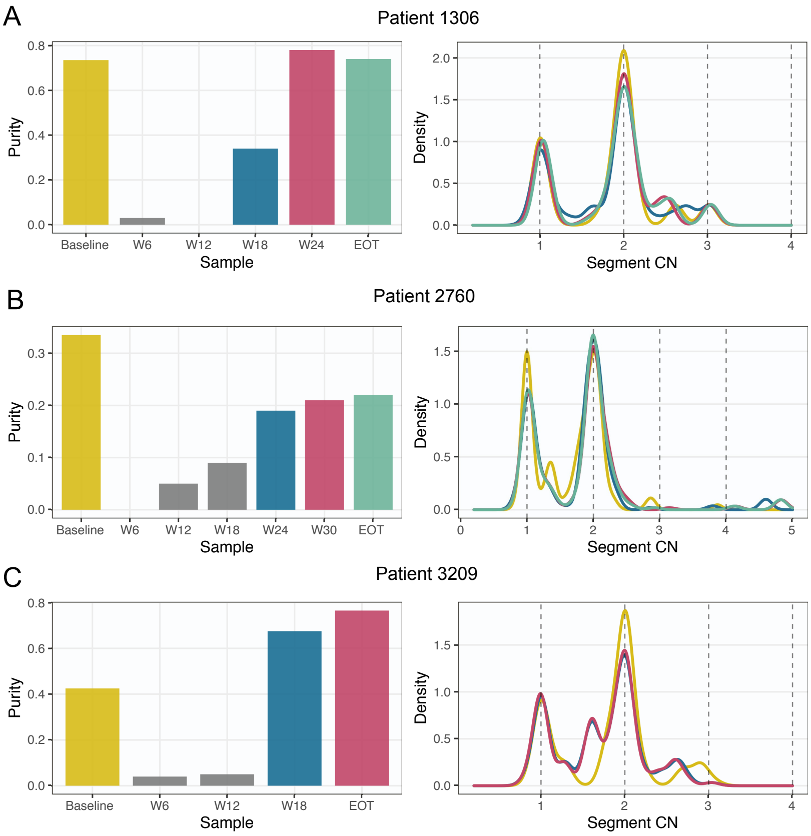

Figure S9: Tumour purity of cfDNA samples from lung cancer patients 1306 (A), 2760 (B), and 3209 (C); related to Fig. 5. The left panel shows the purity estimate for each patient sample. Samples removed from downstream analysis are shown in grey. The right panel shows the purity-corrected CN distribution of non-discarded samples, coloured according to the bars on the left. W: Week, EOT: End-of-therapy.

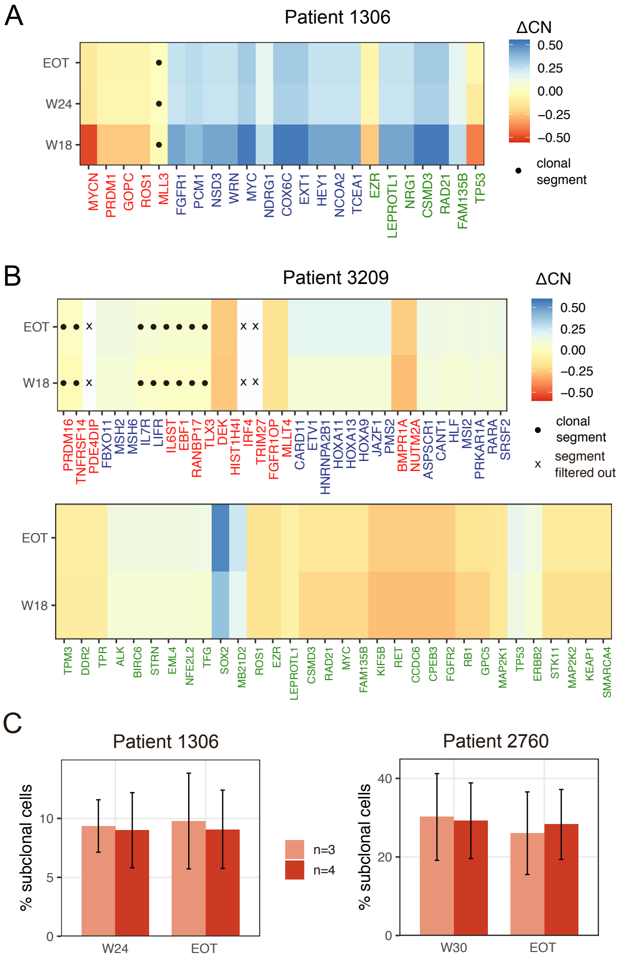

Figure S10: Details of liquidCNA results on *in vivo* cfDNA samples, related to Fig. 5. (A-B) Copy number of the cancer driver genes identified by (Chen et al., 2019) as showing therapy-specific SCNAs in patients 1306 (A) and 3209 (B). Gene names are coloured according to the original study: end-of-therapy gains are in blue, losses are in red, genes newly identified in liquidCNA are in green. The heatmaps represent purity-corrected ΔCN values derived from liquidCNA compared to the baseline sample. Points show genes that fall in genomic segments of clonal SCNAs (dots) or regions filtered out in the quality control step (crosses). (C) Estimated subclonal proportion of the two final time-points from patients 1306 and 2760, computed using all samples of a patient ($n = 4$, dark red) or only these selected time-points ($n = 3$, salmon). W: Week, EOT: End-of-therapy.
